# Associations of Plasma Gut Microbiota-Derived TMAO and Precursors in Early Pregnancy with Gestational Diabetes Mellitus Risk: A Nested Case-Control Study

**DOI:** 10.3390/nu17050810

**Published:** 2025-02-26

**Authors:** Yani Wu, He Bai, Ying Lu, Ruiheng Peng, Mingxia Qian, Xuchen Yang, Enmao Cai, Wenli Ruan, Qianlong Zhang, Jun Zhang, Liqiang Zheng, on behalf of the Shanghai Birth Cohort

**Affiliations:** 1 School of Public Health, Shanghai Jiao Tong University School of Medicine, Shanghai 200025, China; soulnsn@sjtu.edu.cn (Y.W.); bh17737151922@163.com (H.B.);; 2 Department of Physical and Chemical, Changning District Center for Disease Control and Prevention, Shanghai 200050, China; 3 Ministry of Education-Shanghai Key Laboratory of Children’s Environmental Health, Xinhua Hospital, Shanghai Jiao Tong University School of Medicine, Shanghai 200092, China

**Keywords:** trimethylamine N-oxide, choline, betaine, carnitine, gestational diabetes mellitus

## Abstract

**Objectives:** Gut microbiota-derived metabolites—trimethylamine N-oxide (TMAO) and its precursors choline, betaine, and carnitine—have been linked to various health outcomes. However, their role in gestational diabetes mellitus (GDM) remains unclear due to inconsistent findings. This study aims to investigate the associations between maternal plasma concentrations of these metabolites during early pregnancy and the risk of GDM. **Methods:** A nested case–control study was performed in the Shanghai Birth Cohort. GDM cases and non-GDM controls were matched according to maternal age at a ratio of 1:4. Three hundred twenty-one identified GDM cases and 1284 controls were included. Maternal plasma concentrations of TMAO and its precursors were measured between 12 and 16 weeks of gestation in early pregnancy using high-performance liquid chromatography-tandem mass spectrometry. Conditional logistic regression models were applied to assess associations between metabolite levels and GDM risk and to calculate odds ratios (ORs) and their 95% confidence intervals (CIs). Multivariate linear regressions evaluated relationships between metabolite concentrations and glycemic indicators. Stratified and sensitivity analyses were conducted to ensure robustness. **Results:** Maternal plasma levels of TMAO, choline, betaine, and carnitine in early pregnancy were 1.95 μmol/L (IQR, 1.16–3.20), 9.25 μmol/L (IQR, 7.31–11.98), 20.51 μmol/L (IQR, 16.92–24.79), and 17.13 μmol/L (IQR, 13.33–21.16), respectively. Betaine and carnitine were significantly higher in GDM cases (*p* = 0.002 and *p* = 0.042, respectively). No significant associations were identified between TMAO levels and GDM risk and glycemic indicators. Each SD increase in choline was associated with a 16% higher GDM risk (OR = 1.16, 95% CI: 1.01, 1.34, *p* = 0.039), while increased betaine and carnitine levels were linked to a 19% (OR = 0.81, 95% CI: 0.70, 0.95; *p* = 0.010) and 20% (OR = 0.80, 95% CI: 0.69, 0.94; *p* = 0.007) lower risk, respectively. Restricted cubic spline models showed no evidence of non-linear relationships (*p*_for non-linearity_ > 0.05). Interaction analyses indicated that the protective effect of betaine may be more pronounced in parous women. **Conclusions:** Higher early pregnancy levels of betaine and carnitine were associated with a reduced GDM risk, while elevated choline levels increased the risk. The protective association between betaine and GDM was more pronounced in parous women. No significant relationship was found between TMAO and GDM. The roles of choline, betaine, and carnitine in glucose metabolism warrant further investigation.

## 1. Background

Gestational diabetes mellitus (GDM) is a common complication of pregnancy, defined by the onset of glucose intolerance first diagnosed during gestation [1]. Its global incidence has surged in recent years, emerging as a significant public health challenge [2,3]. A meta-analysis reports that the average prevalence of GDM in mainland China is 14.8% [4]. Women with GDM are at heightened risk for pregnancy-related complications, such as hypertension, preeclampsia, preterm birth, and obstructed labor, as well as long-term risks, like type 2 diabetes and cardiovascular disease [5,6]. Beyond maternal health, GDM has profound implications for offspring, increasing the likelihood of childhood obesity, insulin resistance, and metabolic syndrome, which persist into adulthood [7]. Studies have shown that children born to mothers with GDM have an elevated risk of type 2 diabetes and cardiovascular disease later in life [8]. Therefore, identifying novel biomarkers for early GDM prediction and intervention is crucial in obstetrics and gynecology, as early detection and timely management are essential for improving maternal and fetal outcomes while reducing long-term health risks for both mother and child.

Among the identified risk factors for GDM, dietary influences have drawn considerable interest. Recent research underscores the role of specific nutrients in GDM development [9,10]. Trimethylamine N-oxide (TMAO), an organic amine oxide derivative with the molecular formula (CH3)3NO, is primarily produced by gut microbiota and has emerged as a key metabolite of interest [11,12]. Clinical cohort studies have implicated TMAO in developing type 2 diabetes, highlighting its significance in metabolic research [13]. Dietary sources rich in choline, phosphatidylcholine, and L-carnitine—such as red meat, dairy, eggs, and saltwater fish—contribute to TMAO production. Choline-trimethylamine (TMA) lyase, a microbial enzyme, catalyzes the conversion of these dietary compounds into TMA, which is absorbed into the portal circulation and converted into TMAO by flavin-containing monooxygenase 3 (FMO3) in the liver [14,15,16] (Figure S1). The concentration of TMAO is modulated by diet, gut microbiota composition, enzyme activity, gut–liver axis integrity, and hepatic function [17].

Emerging evidence links TMAO and its precursors—choline, betaine, and carnitine—to glucose homeostasis [9,18]. Mechanistically, TMAO disrupts insulin signaling by activating inflammatory pathways, such as the NLRP3 inflammasome, which impairs insulin receptor substrate-1 (IRS-1)-mediated signaling, leading to insulin resistance and glucose intolerance [19]. Furthermore, TMAO promotes adipose tissue inflammation by increasing macrophage infiltration and pro-inflammatory cytokine secretion, exacerbating metabolic dysfunction [13,20]. Our previous studies have identified a significant correlation between serum TMAO levels and type 2 diabetes mellitus (T2DM) risk, suggesting that normalizing serum TMAO through dietary interventions may prevent T2DM [21]. Although the focus is primarily on TMAO, its precursors—choline, betaine, and carnitine—also play crucial roles in glucose metabolism and may serve as potential biomarkers for GDM. Choline is essential for phospholipid synthesis and one-carbon metabolism, and its deficiency has been linked to insulin resistance and an increased risk of metabolic disorders [12]. Betaine has been shown to improve insulin sensitivity and protect pancreatic β-cell function [22]. Carnitine, which facilitates mitochondrial fatty acid oxidation, is essential for maintaining energy homeostasis, and its depletion has been associated with impaired glucose metabolism and metabolic syndrome [23]. Given their metabolic significance, further research is needed to elucidate their precise roles in GDM development and their utility as predictive biomarkers. In studies investigating TMAO and its precursors in GDM, the results have been inconsistent. Early studies suggested that low mid-pregnancy plasma TMAO and betaine levels predispose women to GDM [24]. Conversely, a Chinese prospective study identified early pregnancy serum TMAO as an independent risk factor for mid-pregnancy GDM [25], while a Canadian study found no significant differences in plasma choline and betaine between GDM cases and controls [26]. These discrepancies may be attributed to variations in dietary patterns, gut microbiota composition, and metabolic adaptations during pregnancy.

A significant limitation of the existing research is the predominance of cross-sectional studies, where blood sample collection and GDM diagnosis occur concurrently, limiting the ability to infer causality. Furthermore, many studies are constrained by small sample sizes, reducing statistical power and generalizability. Methodological limitations also include variations in dietary habits and microbiota profiles across different populations, which can significantly influence TMAO and its precursor levels [27]. Furthermore, dynamic fluctuations in these metabolites throughout pregnancy present challenges in establishing their predictive value for GDM. Given the early stage of research on TMAO and GDM, further investigation through large, diverse, prospective cohort studies is essential to clarify these relationships. Therefore, we conducted a case–control study nested within the prospective Shanghai Birth Cohort (SBC) to evaluate the associations between early pregnancy plasma concentrations of TMAO, choline, betaine, and carnitine and the subsequent risk of GDM in mid-pregnancy.

## 2. Methods

### 2.1. Study Design

SBC recruited women in pre-pregnancy and early pregnancy across four administrative districts (two urban districts, one suburban, and one semi-rural district) in Shanghai from 2013 to 2016. A complete description of the SBC is presented elsewhere [28]. The inclusion criteria included: (1) women aged ≥20 years; (2) women or their husbands registered as Shanghai residents; (3) prenatal care and deliveries scheduled to take place at hospitals participating in the SBC; (4) the family intended to reside in Shanghai for a minimum of two years; (5) willingness to participate in follow-up visits. For this study, the exclusion criteria encompassed prior diagnoses of diabetes, severe endocrine disorders (Cushing’s syndrome, hyperthyroidism, and hypothyroidism), or cancer, and the unavailability of oral glucose tolerance test (OGTT) results. Women provided blood samples during early pregnancy, between 12 and 16 weeks of gestation. They were invited for the second study visit at 24–28 weeks gestation. For each GDM case, we selected 4 controls matched by maternal age at pregnancy. Finally, we included 321 GDM cases and 1284 matched non-GDM controls. Consequently, 1605 participants were finally included in this nested case–control study. The flowchart of the study design is shown in Figure S2. All the collection and treatment of biological samples met the standard operating procedures.

This study received approval from the Institutional Review Boards of Xinhua Hospital, affiliated with Shanghai Jiao Tong University School of Medicine. At the study’s initiation, all pregnant participants were provided with detailed information regarding its objectives and procedures, after which they signed an informed consent form.

### 2.2. Diagnosis of Gestational Diabetes Mellitus

GDM was diagnosed based on a 75 g OGTT between 24 and 28 gestational weeks according to the International Association of Diabetes and Pregnancy Study Groups’ (IADPSG) criteria [29] in which the glucose concentration had to meet any of the following 3 thresholds: (a) fasting plasma glucose (FPG) ≥ 5.1 mmol/L; (b) 1 h plasma glucose (1 h-PG) ≥ 10.0 mmol/L; or (c) 2 h plasma glucose (2 h-PG) ≥ 8.5 mmol/L. Blood glucose detection is carried out by an automatic blood glucose meter. Participants were scheduled for their second study visit to coincide with their OGTT appointment.

### 2.3. Determination of Plasma TMAO and Its Precursors

Blood plasma samples were collected between 12 and 16 weeks of gestation from 2013 to 2016 and were stored at −80 °C until analysis in 2023. Before testing, samples were thawed at 4 °C to ensure stability. The concentrations of TMAO and its precursors (choline, betaine, and carnitine) in early pregnancy plasma were quantified using high-performance liquid chromatography-tandem mass spectrometry (HPLC-MS/MS, Shimadzu, Japan). A 10 μL plasma sample was diluted 50 times with the standard solution, followed by vortex mixing and centrifugation at 15,000 r/min for 15 min at 4 °C. The resulting supernatant was passed through a 0.22 μm hydrophobic nylon membrane, and a 100 μL aliquot of the filtered solution was transferred into a sealed sample vial for analysis. Detailed experimental procedures and instrument settings are provided in the supplementary section. The HPLC-MS/MS method developed by our team is characterized by high speed, precision, and sensitivity, meeting the demands of large-scale blood sample analysis.

### 2.4. Measurement of Other Variables

Demographic and pregnancy-related factors were collected using questionnaires administered by trained researchers. These data included participants’ age, race, educational level, pre- and early-pregnancy body mass index (BMI), smoking and alcohol consumption, physical activity, sleep quality, and medical history. Physical activity levels at early-pregnancy were evaluated using the International Physical Activity Questionnaire [30], while the Pittsburgh Sleep Quality Index [31] was used to assess sleep quality at early- pregnancy. Biochemical test indicators were obtained from the participants’ medical records.

### 2.5. Statistical Analysis

The normality of data distribution was assessed using the Kolmogorov–Smirnov test. Descriptive statistics for studied variables are presented as mean  ±  standard deviation (SD) for normally distributed variables, median (interquartile range (IQR) for non-normally distributed variables, and frequency (percentage) for categorical variables. Missing values were multiply imputed using the “chained equations” method.

Conditional logistic regression models were used to calculate odds ratios (ORs) with 95% confidence intervals (CIs) for the associations of the maternal plasma concentrations of TMAO and its precursors (choline, betaine, and carnitine) in early pregnancy (both as categorical and continuous variables) with GDM risk in mid-pregnancy. ORs and corresponding 95% CIs were reported across quartiles of the metabolite concentrations and per SD increment of metabolite concentrations. Covariates were adjusted in multivariable models. The crude model is unadjusted. In the multivariable-adjusted model, we adjusted for age, race, educational level, early pregnancy BMI, physical activity, smoking, alcohol, passive smoke, early pregnancy sleep score, parity, disease history, and the levels of the other three metabolites. These covariates were selected based on the prior literature and biological plausibility. Restricted cubic spline models [32] with four knots were used to explore the potential non-linear associations of the relationships between TMAO and its precursors, and GDM, with adjustment for the covariables in the multivariable-adjusted model. This approach allows for a more detailed exploration of exposure-response relationships without imposing restrictive linearity assumptions. Conditional logistic regression models and interaction tests performed by adding a cross-product term were conducted in stratified analysis by age (<30 years and ≥30 years), BMI (<24.0 kg/m^2^ and ≥24.0 kg/m^2^), and parity (nulliparous and parous).

To further explore the relationship between metabolite concentrations and glucose metabolism, we conducted additional analyses examining their associations with three OGTT-derived glycemic indicators: FPG, 1 h-PG, and 2 h-PG. Multivariate linear regressions were used to estimate the effect, and β-coefficients and corresponding 95% CIs were reported per SD increment of four metabolites’ concentrations and across quartiles of the parameters. Additionally, we conducted a sensitivity analysis using propensity score calculations based on more factors, including age, race, educational level, and parity, to match non-GDM controls and confirm the robustness of our results.

All statistical analyses were performed using R Studio version 4.1.2 (PBC, Boston, MA, USA). To account for multiple comparisons, we applied the False Discovery Rate (FDR) correction using the Benjamani–Hochberg method. Two-sided *p* < 0.05 was considered statistically significant. In contrast, *p* < 0.15 was considered statistically significant for the interaction effect analyses.

## 3. Results

We included 321 cases of GDM and 1284 matched controls. The baseline demographic and biochemical characteristics of the participants are shown in Table 1. The mean age of women at recruitment was 29.7 ± 5.2 years old, with a median pre-pregnancy BMI of 21.0 kg/m^2^. Maternal plasma levels of TMAO, choline, betaine, and carnitine in early pregnancy were 1.95 μmol/L (IQR, 1.16–3.20), 9.25 μmol/L (IQR, 7.31–11.98), 20.51 μmol/L (IQR, 16.92–24.79), and 17.13 μmol/L (IQR, 13.33–21.16), respectively. Compared to controls, GDM cases exhibited significantly higher pre- and early-pregnancy BMI (*p* < 0.001). No significant differences were observed between cases and controls regarding ethnicity, education level, physical activity, smoking status, alcohol consumption, or sleep quality (*p* > 0.05). Glycemic indicators derived from OGTT, including FPG, 1 h-PG, and 2 h-PG, were significantly elevated in the GDM group compared to controls (*p* < 0.001). While plasma concentrations of TMAO and choline did not differ significantly between the two groups (*p* > 0.05), betaine levels were notably higher in the GDM cases (*p* = 0.002), as were carnitine levels (*p* = 0.042). Significant partial correlations were observed between plasma levels of TMAO, choline, betaine, and carnitine (*p* < 0.001)—except for TMAO versus carnitine and betaine versus carnitine (Table S1).

**Figure 1:**
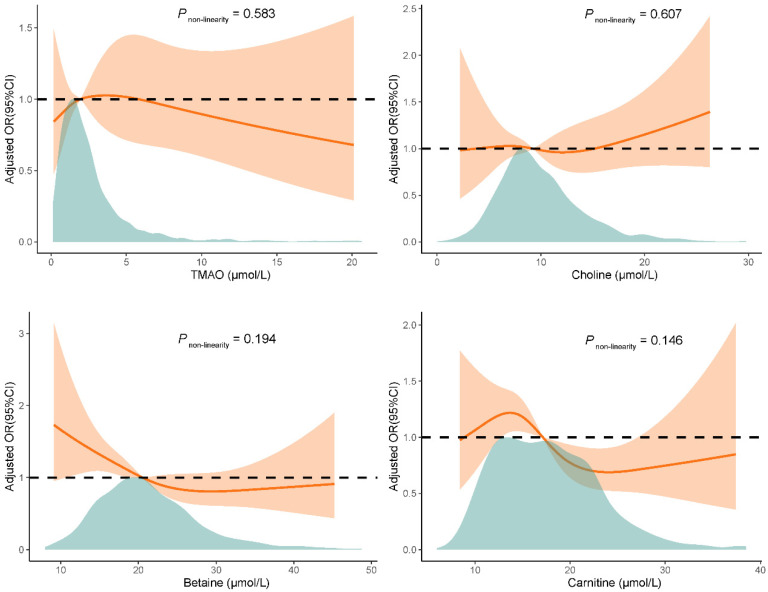
Multivariable-adjusted association of TMAO and its precursors with risk of GDM by restricted cubic regression. The green shaded area indicates the distribution of the four metabolites (TMAO, choline, betaine, carnitine). The solid lines show odd ratios, and the orange-shaded area exhibits 95% confidence intervals (CI). Estimates were adjusted for age, race, educational level, early pregnancy BMI, physical activity, smoking, alcohol, passive smoke, early pregnancy sleep score, pregnancy history, disease history, and the levels of the other three metabolites. Abbreviations: TMAO, trimethylamine N-oxide.

Then, we examined the association between four metabolites and GDM risk. As shown in Table 2, significant associations were observed between the precursors—choline, betaine, and carnitine—with the risk of GDM, in multivariable-adjusted models as continuous variables. Specifically, for each standard deviation (SD) increase in choline levels, the odds of developing GDM increased by 16% in the multivariate-adjusted model (OR = 1.16, 95% CI: 1.01, 1.34, *p* = 0.039). In contrast, lower risks of GDM were observed with an increased level of betaine and carnitine, per SD increase in betaine and carnitine were associated with a 19% (OR = 0.81, 95% CI: 0.70, 0.95; *p* = 0.010) and 20% (OR = 0.80, 95% CI: 0.69, 0.94; *p* = 0.007) decrease in the risk of GDM. When analyzing metabolite concentrations by quartiles and comparing them to the lowest quartile, the estimates for the risk in the second and fourth quartiles of betaine were 0.65 (Q2 vs. Q1, 95% CI: 0.44, 0.96, *p* = 0.029) and 0.49 (Q4 vs. Q1, 95% CI: 0.32, 0.75, *p* = 0.001), respectively. Similarly, compared with the lowest quartile of carnitine, both the third quartile (Q3 vs. Q1, OR = 0.62, 95% CI: 0.41, 0.94, *p* = 0.024) and the fourth quartile (Q4 vs. Q1, OR = 0.47, 95% CI: 0.30, 0.74, *p* = 0.001) of maternal plasma carnitine levels were associated with a decreased risk of GDM (per SD increment, OR = 0.83, 95% CI: 0.73, 0.95; *p* = 0.008). However, no significant associations were identified between TMAO levels and GDM risk, whether assessed as continuous variables or by quartiles. Furthermore, as depicted in Figure 1, restricted cubic spline models revealed no evidence of non-linear associations between any of the four metabolites and GDM risk (*P*_for non-linearity_ > 0.05).

**Figure 2:**
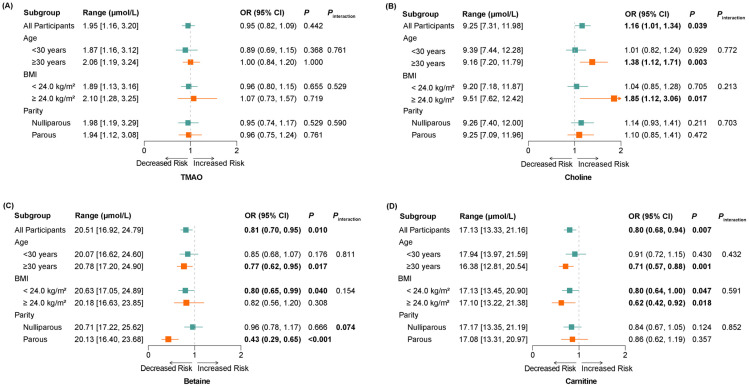
Stratified analyses of the association between TMAO and its precursors with the risk of GDM. Conditional logistic regression models were used to estimate the associations, adjusting for age, race, educational level, early pregnancy BMI, physical activity, smoking, alcohol, passive smoke, early pregnancy sleep score, pregnancy history, disease history, and the levels of the other three metabolites, excluding the stratifying variables per se. The effect estimate was expressed as odds ratios (OR) and 95% confidence interval (95% CI). (**A**–**D**) show the individual effects of TMAO, Choline, Betaine, and Carnitine respectively, on the risk of GDM. The green and orange squares and lines represent the two groups, respectively. Statistically significant results are bolded. Abbreviations: BMI, body mass index.

Then, we conducted stratified analyses to explore whether the associations between TMAO, its precursors, and GDM were modified by age, BMI, or parity. As shown in Figure 2, the associations between choline, betaine, and carnitine with GDM appeared more pronounced among women aged ≥30 years. The BMI-stratified analysis revealed distinct patterns in the associations between metabolite concentrations and GDM. Choline demonstrated a significant positive association with GDM in women with BMI ≥ 24.0 kg/m^2^ (OR = 1.85, 95% CI: 1.12, 3.06; *p* = 0.017), whereas betaine was inversely associated with GDM in the lower BMI group (<24.0 kg/m^2^) (OR = 0.80, 95% CI: 0.65, 0.99; *p* = 0.040). Carnitine exhibited a consistent negative association with GDM across both BMI categories (*p* < 0.05). However, interactions between these metabolites and age or BMI did not reach statistical significance (*P*_interaction_ > 0.15). Notably, a significant interaction between betaine and parity was observed (*P*_interaction_ < 0.05). In parous women, each SD increase in betaine concentration was associated with a 57% reduction in GDM risk (OR = 0.43, 95% CI: 0.29, 0.65; *p* < 0.001), whereas no significant association was detected in nulliparous women.

Table 3 presents the associations between maternal concentrations of TMAO and its precursors in early pregnancy and glycemic indicators in mid-pregnancy. After adjusting for confounders as described in the Section 2, maternal plasma choline levels in early pregnancy were positively associated with 1 h-PG levels (Q2 vs. Q1, β = 0.28, 95% CI: 0.05, 0.51; *p* = 0.017). In contrast, betaine and carnitine demonstrated inverse correlations with glycemic indicators. Notably, each 1 SD increase in carnitine concentration was associated with a lower 2 h-PG level (β = −0.08, 95% CI: −0.15, 0.00; *p* = 0.048). Betaine exhibited consistent inverse associations across all three glycemic indicators—FPG, 1 h-PG, and 2 h-PG—whether analyzed as continuous variables or by quartiles. For example, each 1 SD increment in betaine concentration was associated with significant reductions in FBG (β = −0.03, 95% CI: −0.06, −0.01; *p* = 0.016) and 2 h-PG (β = −0.10, 95% CI: −0.17, −0.02; *p* = 0.010). Similar trends were observed when comparing quartiles, with participants in the highest quartile of betaine showing the most pronounced decreases in glycemic indicators compared to those in the lowest quartile. These findings underscore the potential protective role of betaine in glucose metabolism during pregnancy. Consistent with the findings for GDM, TMAO showed no significant associations with any OGTT indicators in the adjusted models (*p* > 0.05).

A propensity score calculation based on more factors analysis was further employed for the sensitivity analysis. Table S2 presents the baseline demographic and biochemical characteristics of the newly matched cases and controls. The associations between the three precursors and GDM risk, as well as glucose indicators, remained consistent, which confirmed the robustness of our findings (Tables S3 and S4).

## 4. Discussion

This study examined the plasma concentrations of TMAO and its precursors—choline, betaine, and carnitine—in early pregnancy and their associations with the risk of GDM. While no significant relationship was found between plasma TMAO levels and GDM risk, plasma betaine and carnitine levels were lower in the GDM group compared to the controls. Higher levels of betaine and carnitine were inversely associated with GDM risk, whereas elevated choline levels were positively correlated with an increased risk of GDM. Our evaluation of potential effect modifiers indicated that the associations between metabolite levels and GDM risk were generally consistent across maternal age, BMI, and parity, except for betaine, where the protective association was more pronounced in parous women than nulliparous women. The stronger associations observed in women ≥30 years of age and in specific BMI categories may reflect age- and BMI-related differences in metabolic regulation. Older women and those with higher BMI are more likely to exhibit insulin resistance and altered gut microbiota composition, which could amplify the effects of betaine and carnitine on glucose metabolism. These findings are consistent with previous studies showing that age and obesity are significant risk factors for GDM [33]. However, the interaction between these factors and metabolite levels raises new research questions about how metabolic pathways and gut microbiota profiles vary across different subpopulations, warranting further investigation.

TMAO is an organic molecule produced by the gut microbiota from dietary choline, betaine, and carnitine [34]. TMAO is closely related to gut microbiota and dietary intake [35]. Animal studies suggest that TMAO can increase blood glucose levels and disrupt lipid homeostasis by inhibiting FMO3 [36], a key enzyme involved in its metabolism. However, findings from human studies are mixed. Some studies have found no significant association between TMAO and diabetes or GDM [23,37,38,39] while others report that TMAO may be an independent risk factor for GDM [6,40,41], and some even observe a negative association between TMAO and GDM [24,42]. Several case–control studies in pregnant populations did not find an association between serum TMAO and GDM [23,37]. A twin-pregnancy cohort study in China also found no significant link between serum TMAO and GDM risk [39]. Furthermore, Mendelian randomization studies have not found a causal relationship between TMAO and GDM [23]. These findings align with ours, where we did not observe a significant association between TMAO levels and GDM risk. This suggests that TMAO may not play a direct causal role in GDM development, or its effects may be masked by other factors such as gut microbiota composition or dietary intake. However, the inconsistent findings across studies highlight the need for further research to clarify the role of TMAO in GDM pathophysiology. A prospective study from a Boston-based birth cohort involving 1496 pregnant women showed a positive correlation between maternal plasma TMAO levels and the risk of GDM [41]. Similarly, a nested case–control study in China, including 220 GDM cases and 220 controls, demonstrated that serum TMAO levels were higher in GDM women than controls, with TMAO levels identified as an independent risk factor for GDM [6]. These differences may be attributed to factors such as FMO3 enzyme activity, gut microbiota composition, and dietary intake. TMAO production depends on the metabolism of dietary precursors by the gut microbiota, and differences in diet and microbiota could influence TMAO production, thereby affecting glucose metabolism [37].

Our study shows that the precursors of TMAO (choline, betaine, and carnitine) have a more significant role in GDM risk than TMAO itself. This may be because these precursors directly affect insulin signaling pathways or glucose metabolism regulation. Choline is closely related to insulin resistance and hyperglycemia. The present study found a positive association between choline levels and the risk of GDM. This finding is supported by both mechanistic evidence and population-based studies. Mechanistically, animal studies have shown that choline may promote insulin resistance and hyperglycemia in the liver of mice with impaired phosphatidylethanolamine N-methyltransferase function [6,12]. Furthermore, a nested case–control study found that higher choline levels in early pregnancy were associated with increased GDM risk [6]. These consistent findings across different study designs and populations strengthen the validity of our results and highlight the potential role of choline in the pathogenesis of GDM.

In contrast, the present study identified betaine and carnitine as protective factors against GDM. The biological mechanisms underlying their effects on glucose metabolism are well-supported by existing evidence. Betaine, as an osmolyte and methyl donor, may improve insulin sensitivity by activating AMPK and modulating methylation pathways, which are critical for glucose homeostasis [22]. Carnitine, on the other hand, plays a pivotal role in fatty acid oxidation and mitochondrial function. By facilitating the transport of long-chain fatty acids into mitochondria for β-oxidation, carnitine helps maintain energy balance and reduces lipid accumulation, thereby improving insulin sensitivity and glucose metabolism [23]. These mechanisms are consistent with population-based studies, such as a twin-pregnancy cohort study that reported a negative correlation between serum betaine levels and GDM risk [38], and a meta-analysis also confirmed that carnitine improves glucose metabolism in diabetic patients [43]. A study investigating the relationship between serum carnitine-TMA-TMAO metabolism and GDM found a negative correlation between serum L-carnitine in mid-pregnancy and GDM risk, with L-carnitine being an independent protective factor for GDM [37]. These studies may explain the protective associations of betaine and carnitine with GDM risk observed in our study. The composition of the gut microbiota also plays a significant role in modulating the levels of these metabolites and their subsequent metabolic effects during pregnancy. For instance, gut bacteria such as *Bifidobacterium* and *Lactobacillus* are involved in the metabolism of choline and carnitine, which can influence the production of TMAO and other intermediates [37]. Variations in gut microbiota composition, driven by factors such as diet, age, and hormonal changes during pregnancy, may alter the levels of betaine and carnitine, thereby affecting glucose metabolism and GDM risk. This highlights the potential interplay between gut microbiota, dietary precursors, and metabolic health in pregnancy.

The findings of this study have important implications for potential early screening and intervention strategies for GDM. Measurement of betaine and carnitine levels in early pregnancy could serve as valuable biomarkers for identifying women at higher risk of GDM. For example, women with low betaine or carnitine levels might benefit from targeted dietary interventions, such as increased intake of betaine-rich foods (e.g., spinach, and beets) or carnitine supplementation. Personalized nutritional strategies could be developed based on individual metabolite profiles, potentially reducing GDM risk and improving pregnancy outcomes. However, it is important to clarify that plasma levels of TMAO, choline, betaine, and carnitine do not directly reflect the dietary intake of these compounds. Although our findings suggest an association between elevated plasma choline concentrations and an increased risk of GDM, recommending dietary restrictions or alterations based solely on these results would be premature. The body’s demand for choline of women increases during pregnancy. Current evidence indicates that most pregnant women fail to meet the recommended intake levels for this nutrient [44]. Further investigations are necessary to assess whether early dietary modifications for choline during pregnancy could mitigate GDM risk.

Our study has several strengths. First, we extensively evaluated metabolites involved in the TMAO biosynthesis pathway. Beyond assessing the gut microbiota-derived metabolite TMAO, we also investigated its three key precursors, which are rarely measured repeatedly in comparable studies, thereby enhancing the comprehensiveness of our design. Importantly, metabolite measurements were obtained between 12 and 16 weeks of pregnancy, supporting exploring potential causal relationships. The large sample size and the nested case–control design also provided robust and reliable results with sufficient statistical power. However, several limitations should be considered when interpreting our results. Our study did not include detailed dietary data for pregnant women, limiting the ability to account for dietary factors that could influence the results. Although the primary aim of this study was to assess the associations between TMAO and its precursors with GDM, our ability to evaluate the modifying roles of diet and gut microbiota was constrained by the absence of both dietary intake data and microbiome measurements. Given that dietary intake can influence plasma levels of TMAO precursors, this may partially confound observed associations. Future research should explore dietary interventions and gut microbiota modulation to further elucidate their effects on TMAO and its precursors, contributing to evidence for early screening and potential intervention strategies for GDM. Furthermore, as TMAO, choline, betaine, and carnitine are in the same metabolic pathway, regression models that examine each metabolite independently or adjust may not fully capture the biological complexity of these associations. Applying more advanced methodologies could better evaluate these interrelationships and provide valuable insights for future investigations.

## 5. Conclusions

In conclusion, this study found that higher levels of betaine and carnitine in early pregnancy were associated with a reduced risk of GDM, while elevated choline levels were linked to an increased risk. Additionally, the protective association between betaine and GDM was more pronounced in parous women than in nulliparous women. Although we did not find a significant relationship between TMAO and GDM risk, the role of choline, betaine, and carnitine in glucose metabolism appears more prominent. This suggests that diet and gut microbiota may influence the onset and development of GDM by regulating the levels of these metabolites. Future research should explore the mechanisms of action of TMAO and its precursors and evaluate the potential of dietary interventions to reduce GDM risk. These studies may provide new insights and strategies for preventing and managing GDM.

## Figures and Tables

**Table 1:** Participant characteristics.

	Overall (N = 1605)	Controls (N = 1284)	GDM Cases (N = 321)	*p*
**Prenatal characteristics**				
Age (years)	29.7 ± 5.2	29.7 ± 5.3	29.9 ± 5.1	0.994
Ethnicity				0.549
Han	1594 (99.3)	1276 (99.4)	318 (99.1)	
Others	11 (0.7)	8 (0.6)	3 (0.9)	
Maternal education				0.134
High school and below	142 (8.8)	106 (8.3)	36 (11.2)	
College	390 (24.3)	312 (24.3)	78 (24.3)	
University	858 (53.5)	691 (53.8)	167 (52.0)	
Postgraduate degree	215 (13.4)	175 (13.6)	40 (12.5)	
Pre-pregnancy BMI (kg/m^2^)	21.00 [19.50, 23.10]	20.80 [19.20, 22.80]	22.00 [20.20, 24.70]	**<0.001**
Early-pregnancy BMI (kg/m^2^)	22.00 [20.30, 24.20]	21.80 [20.20, 23.80]	23.40 [21.10, 26.20]	**<0.001**
Physical activity				0.694
Low	709 (44.2)	572 (44.5)	137 (42.7)	
Middle	859 (53.5)	681 (53.0)	178 (55.5)	
High	37 (2.3)	31 (2.4)	6 (1.9)	
Smoke	38 (2.4)	29 (2.3)	9 (2.8)	0.555
Alcohol consumptions	195 (12.1)	152 (11.8)	43 (13.4)	0.439
Live with smokers	551 (34.3)	437 (34.0)	114 (35.5)	0.611
Early pregnancy sleep	4.00 [3.00, 6.00]	4.00 [3.00, 6.00]	4.00 [3.00, 6.00]	0.271
Parity				0.848
Nulliparous	962 (59.9)	771 (60.0)	191 (59.5)	
Parous	643 (40.1)	513 (40.0)	130 (40.5)	
History of gestational hypertension	3 (0.2)	2 (0.2)	1 (0.3)	0.571
History of GDM	18 (1.1)	5 (0.4)	13 (4.0)	**<0.001**
History of hypertension	3 (0.2)	0 (0.0)	3 (0.9)	0.994
History of hyperlipidemia	17 (1.1)	11 (0.9)	6 (1.9)	0.124
Other disease	366 (22.8)	309 (24.1)	57 (17.8)	**0.014**
TMAO (μmol/L)	1.95 [1.16, 3.20]	1.95 [1.15, 3.20]	2.01 [1.21, 3.23]	0.734
Choline (μmol/L)	9.25 [7.31, 11.98]	9.23 [7.26, 11.99]	9.40 [7.64, 11.97]	0.173
Betaine (μmol/L)	20.51 [16.92, 24.79]	20.63 [17.22, 25.02]	19.10 [15.74, 23.42]	**0.002**
Carnitine (μmol/L)	17.13 [13.33, 21.16]	17.39 [13.47, 21.34]	16.28 [13.07, 20.44]	**0.042**
FPG (mmol/L)	4.40 [4.14, 4.70]	4.30 [4.10, 4.60]	4.80 [4.40, 5.20]	**<0.001**
1 h PG (mmol/L)	7.80 [6.70, 8.80]	7.47 [6.52, 8.30]	10.00 [8.60, 10.60]	**<0.001**
2 h PG (mmol/L)	6.50 [5.70, 7.40]	6.30 [5.59, 7.00]	8.50 [7.30, 9.10]	**<0.001**
TG (mmol/L)	1.45 [1.12, 2.24]	1.40 [1.10, 2.16]	1.66 [1.24, 2.57]	**<0.001**
TC (mmol/L)	4.70 [4.20, 5.36]	4.67 [4.17, 5.30]	4.84 [4.28, 5.55]	**0.013**
HDL (mmol/L)	1.18 [1.01, 1.46]	1.17 [1.01, 1.44]	1.22 [1.04, 1.55]	**0.006**
ALT (mmol/L)	14.00 [9.00, 22.00]	14.00 [9.00, 22.00]	16.00 [10.00, 25.00]	**0.016**
AST (mmol/L)	17.00 [14.00, 21.00]	17.00 [14.00, 20.00]	17.00 [14.00, 22.00]	0.079

Data are shown as n (%), mean ± SD, or median [IQR]. The comparison of characteristics between GDM cases and non-GDM controls was conducted using univariate conditional logistic regression. Statistically significant results are bolded. Abbreviations: BMI, body mass index; GDM, gestational diabetes mellitus; TMAO, trimethylamine N-oxide; FPG, fasting plasma glucose; PG, plasma glucose; TG, triglycerides; TC, total cholesterol; HDL, High-density lipoprotein; ALT, alanine aminotransferase; AST, aspartate aminotransferase.

**Table 2:** Associations between maternal trimethylamine N-oxide (TMAO) and its precursor concentrations in early pregnancy and the risk of GDM in mid-pregnancy.

	Crude Model		Multivariable Adjusted Model ^a^	
	OR (95% CI) ^b^	*p*	OR (95% CI)	*p*
**TMAO (μmol/L)**				
Q1 (≤1.16)	Ref.		Ref.	
Q2 (1.16–1.95)	1.15 (0.81, 1.62)	0.440	1.19 (0.82, 1.74)	0.365
Q3 (1.95–3.20)	1.12 (0.79, 1.59)	0.525	1.07 (0.73, 1.58)	0.715
Q4 (>3.20)	1.12 (0.78, 1.61)	0.531	1.11 (0.74, 1.66)	0.616
Continuous ^c^	0.98 (0.86, 1.11)	0.734	0.95 (0.82, 1.09)	0.442
**Choline (μmol/L)**				
Q1 (≤7.31)	Ref.		Ref.	
Q2 (7.31–9.25)	1.28 (0.90, 1.83)	0.170	1.24 (0.83, 1.85)	0.301
Q3 (9.25–11.98)	1.31 (0.91, 1.87)	0.143	1.35 (0.90, 2.03)	0.153
Q4 (>11.98)	1.20 (0.82, 1.74)	0.346	1.41 (0.91, 2.20)	0.124
Continuous	1.09 (0.96, 1.23)	0.173	**1.16 (1.01, 1.34)**	**0.039**
**Betaine (μmol/L)**				
Q1 (≤16.92)	Ref.		Ref.	
Q2 (16.67–20.51)	**0.64 (0.45, 0.91)**	**0.013**	**0.65 (0.44, 0.96)**	**0.029**
Q3 (20.51–24.79)	0.71 (0.50, 1.00)	0.053	0.69 (0.46, 1.03)	0.066
Q4 (>24.79)	**0.46 (0.32, 0.68)**	**<0.001**	**0.49 (0.32, 0.75)**	**0.001**
Continuous	**0.80 (0.70, 0.92)**	**0.002**	**0.81 (0.70, 0.95)**	**0.010**
**Carnitine (μmol/L)**				
Q1 (≤13.33)	Ref.		Ref.	
Q2 (13.33–17.13)	0.95 (0.67, 1.34)	0.770	0.79 (0.53, 1.17)	0.235
Q3 (17.13–21.16)	0.73 (0.51, 1.06)	0.103	**0.62 (0.41, 0.94)**	**0.024**
Q4 (>21.16)	**0.61 (0.42, 0.90)**	**0.013**	**0.47 (0.30, 0.74)**	**0.001**
Continuous	**0.87 (0.75, 0.99)**	**0.042**	**0.80 (0.68, 0.94)**	**0.007**

^a.^ The multivariable model adjusted for age, race, educational level, early pregnancy BMI, physical activity, smoking, alcohol, passive smoke, early pregnancy sleep score, pregnancy history, disease history, and the levels of the other three metabolites. ^b^ Conditional logistic regression was used to estimate the associations. The effect estimate was expressed as odds ratios (OR) and 95% confidence interval (95% CI). Statistically significant results are bolded. ^c.^ “Continuous” refers to the increase in per standard deviation (SD).

**Table 3:** Associations between maternal concentrations of trimethylamine N-oxide (TMAO) and its precursors in early pregnancy and glycemic indicators in mid-pregnancy.

	FPG		1 h PG		2 h PG	
	β (95% CI) ^a^	*p*	β (95% CI)	*p*	β (95% CI)	*p*
**TMAO (μmol/L)**						
Q1 (≤1.16)	Ref.		Ref.		Ref.	
Q2 (1.16–1.95)	0.01 (−0.07, 0.08)	0.869	0.11 (−0.11, 0.34)	0.327	0.03 (−0.17, 0.23)	0.744
Q3 (1.95–3.20)	0.05 (−0.03, 0.12)	0.219	−0.03 (−0.25, 0.2)	0.825	−0.02 (−0.23, 0.18)	0.815
Q4 (>3.20)	0.05 (−0.02, 0.12)	0.194	0.09 (−0.14, 0.32)	0.429	0.07 (−0.14, 0.27)	0.515
Continuous ^b^	0.01 (−0.02, 0.04)	0.427	0.03 (−0.05, 0.11)	0.506	0.03 (−0.05, 0.10)	0.479
**Choline (μmol/L)**						
Q1 (≤7.31)	Ref.		Ref.		Ref.	
Q2 (7.31–9.25)	0.02 (−0.05, 0.09)	0.592	0.03 (−0.19, 0.26)	0.768	0.09 (−0.11, 0.29)	0.391
Q3 (9.25–11.98)	0.06 (−0.02, 0.13)	0.140	**0.28 (0.05, 0.51)**	**0.017**	0.16 (−0.04, 0.37)	0.120
Q4 (>11.98)	−0.02 (−0.10, 0.06)	0.646	0.16 (−0.09, 0.40)	0.212	0.04 (−0.18, 0.25)	0.743
Continuous	−0.01 (−0.04, 0.02)	0.400	0.08 (0.00, 0.17)	0.061	0.03 (−0.04, 0.11)	0.369
**Betaine (μmol/L)**						
Q1 (≤16.92)	Ref.		Ref.		Ref.	
Q2 (16.67–20.51)	−0.03 (−0.10, 0.04)	0.429	−0.13 (−0.36, 0.10)	0.260	−0.07 (−0.27, 0.13)	0.499
Q3 (20.51–24.79)	**−0.07 (−0.15, 0.00)**	**0.047**	−0.03 (−0.26, 0.20)	0.811	−0.16 (−0.36, 0.05)	0.126
Q4 (>24.79)	**−0.11 (−0.19, −0.03)**	**0.004**	**−0.29 (−0.52, −0.05)**	**0.017**	**−0.33 (−0.54, −0.12)**	**0.002**
Continuous	**−0.03 (−0.06, −0.01)**	**0.016**	−0.08 (−0.16, 0.00)	0.063	**−0.10 (−0.17, −0.02)**	**0.010**
**Carnitine (μmol/L)**						
Q1 (≤13.33)	Ref.		Ref.		Ref.	
Q2 (13.33–17.13)	0.03 (−0.04, 0.10)	0.389	0.07 (−0.15, 0.30)	0.530	−0.05 (−0.25, 0.16)	0.659
Q3 (17.13–21.16)	−0.02 (−0.10, 0.05)	0.527	−0.09 (−0.33, 0.14)	0.444	−0.16 (−0.37, 0.04)	0.123
Q4 (>21.16)	0.04 (−0.03, 0.12)	0.270	−0.09 (−0.33, 0.15)	0.477	−0.21 (−0.42, 0.01)	0.058
Continuous	0.02 (0.00, 0.05)	0.087	−0.02 (−0.11, 0.06)	0.615	**−0.08 (−0.15, 0.00)**	**0.048**

^a.^ Multivariate linear regression was used to estimate the associations, adjusting for age, race, educational level, early pregnancy BMI, physical activity, smoking, alcohol, passive smoke, early pregnancy sleep score, pregnancy history, disease history and the levels of other three metabolites. The effect estimate was expressed as a beta coefficient (β) and 95% confidence interval (95% CI). Statistically significant results are bolded. ^b^ “Continuous” refers to the increase in per standard deviation (SD). Abbreviations: TMAO, trimethylamine N-oxide; FPG, fasting plasma glucose; 1 h PG, 1 h plasma glucose; 2 h PG, 2 h plasma glucose.

## Data Availability

The data that support the findings of this study are available from the Shanghai Birth Cohort (SBC), but restrictions apply to the availability of these data, which were used under license for the current study, and so are not publicly available.

## Supplementary Materials

The following supporting information can be downloaded at: https://www.mdpi.com/article/10.3390/nu17050810/s1, Figure S1. Biosynthesis of trimethylamine N-oxide (TMAO) and Its Precursors.; Figure S2. Flow Chart of the Study Participants’ Selection; Table S1. Spearman partial correlation coefficients (ρ); Table S2. Participant characteristics in sensitivity analysis; Table S3. Associations between maternal trimethylamine N-oxide (TMAO) and its precursor concentrations in early pregnancy and the risk of GDM in mid-pregnancy in sensitivity analysis; Table S4. Associations between maternal concentrations of trimethylamine N-oxide (TMAO) and its precursors in early pregnancy and glycemic indicators in mid-pregnancy in sensitivity analysis.
