# Supplementary material for: Associations of Plasma Gut Microbiota-Derived TMAO and Precursors in Early Pregnancy with Gestational Diabetes Mellitus Risk: A Nested Case-Control Study
Source: Nutrients. 2025 Feb 26;17(5):810. doi: 10.3390/nu17050810 (PMC11901518; doi:10.3390/nu17050810)
Supplement: Supplementary file 1 [file nutrients-17-00810-s001.zip › nutrients-3467068-supplementary.pdf]

Supplementary Materials

Methods and settings of HPLC-MS / MS.

Figure S1. Biosynthesis of trimethylamine N-oxide (TMAO) and Its Precursors.

Figure S2. Flow Chart of the Study Participants' Selection.

Table S1. Spearman partial correlation coefficients (ρ).

Table S2. Participant characteristics in sensitivity analysis.

Table S3. Associations between maternal trimethylamine N-oxide (TMAO) and its precursor concentrations in early pregnancy and the risk of GDM in mid-pregnancy in sensitivity analysis.

Table S4. Associations between maternal concentrations of trimethylamine N-oxide (TMAO) and its precursors in early pregnancy and glycemic indicators in mid-pregnancy in sensitivity analysis.

Methods and settings of HPLC-MS / MS.

10μL of plasma was diluted 50 times with the standard, vortexed and mixed, and centrifuged at 4°C and 15000r/min for 15 min, The supernatant was taken and passed through a 0.22μm hydrophobic nylon filter membrane, and finally, 100μL solution was transferred to a sealed sample vial. HPLC-MS/MS was used for detection. The detection limit and quantitative limit of this detection method were 0.003 and 0.062 μmol / L, respectively. The linear range was 0.16~20.00μmol/L (r2=0.999), and the recovery rate was 90.20%~102.10%. The HPLC-MS/MS method for measuring TMAO established by our research team has the characteristics of fast, accurate and sensitive, which can meet the requirements of blood sample detection in large sample populations.

Chromatographic conditions: ACQUITY UPLC HSS T3 column (2.1 mm × 100 mm, 1.7 μm), column temperature 40 °C, injection volume 2 μL. Mobile phase A: 10 mmol/L ammonium formate, 0.1% formic acid, acetonitrile/water = 9:1; mobile phase B: 5 mmol/L ammonium formate, 0.1% formic acid, acetonitrile/water = 1:1. Gradient elution: 0 ~ 2.0 min, mobile phase B was 10%; 2.0 ~ 6.0 min, from mobile phase B 10 % linear change to mobile phase B 45%; 6.0 ~ 6.1 min, mobile phase B 45% linear change to mobile phase B 100%; 6.1 ~ 8.1min, mobile phase B 100 %; 8.1-8.2 min, from mobile phase B 100% back to mobile phase B 10 %; 8.2-10.0 min, mobile phase B 10 %. The flow rate was 0.4 mL/min (split ratio 5: 3).

Mass spectrometry conditions: Electrospray ionization source (ESI), impact voltage 20 eV, positive ion mode, multiple reaction monitoring (MRM), drying gas flow rate 3 L / min, atomizer pressure 50 psi, dryer temperature 350 °C, capillary voltage 3500 V.

Detection effect: When the impact voltage was set to 20 eV and the quantitative ion pair was determined to be m / z 76.1 → 58.1, the detection limit and quantitative limit of this detection method were 0.003 and 0.062 μmol / L, respectively. The linear range was 0.16 ~ 20.00 μmol / L (r2 = 0.999), and the recovery rate was 90.20% ~ 102.10%.

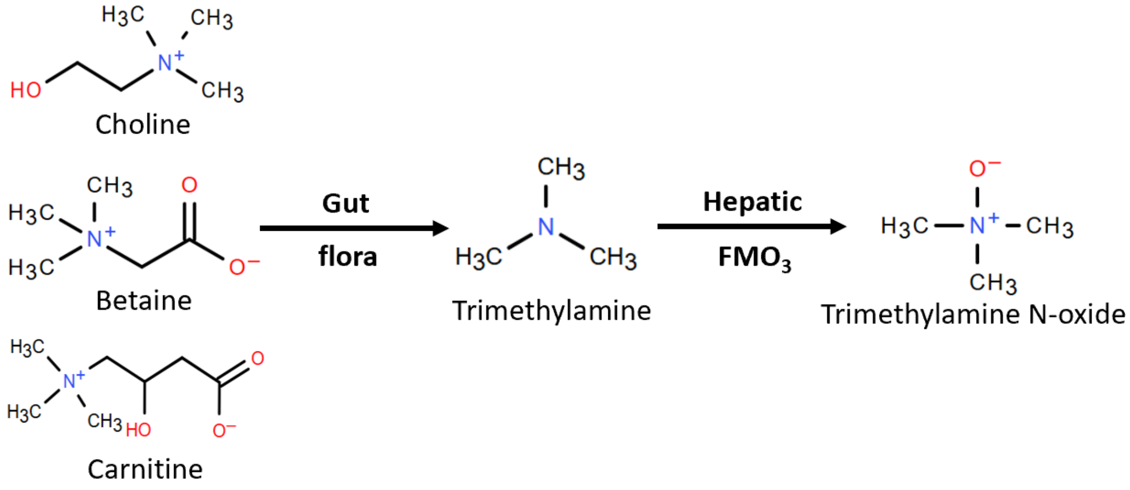

Figure S1. Biosynthesis of trimethylamine N-oxide (TMAO) and Its Precursors.

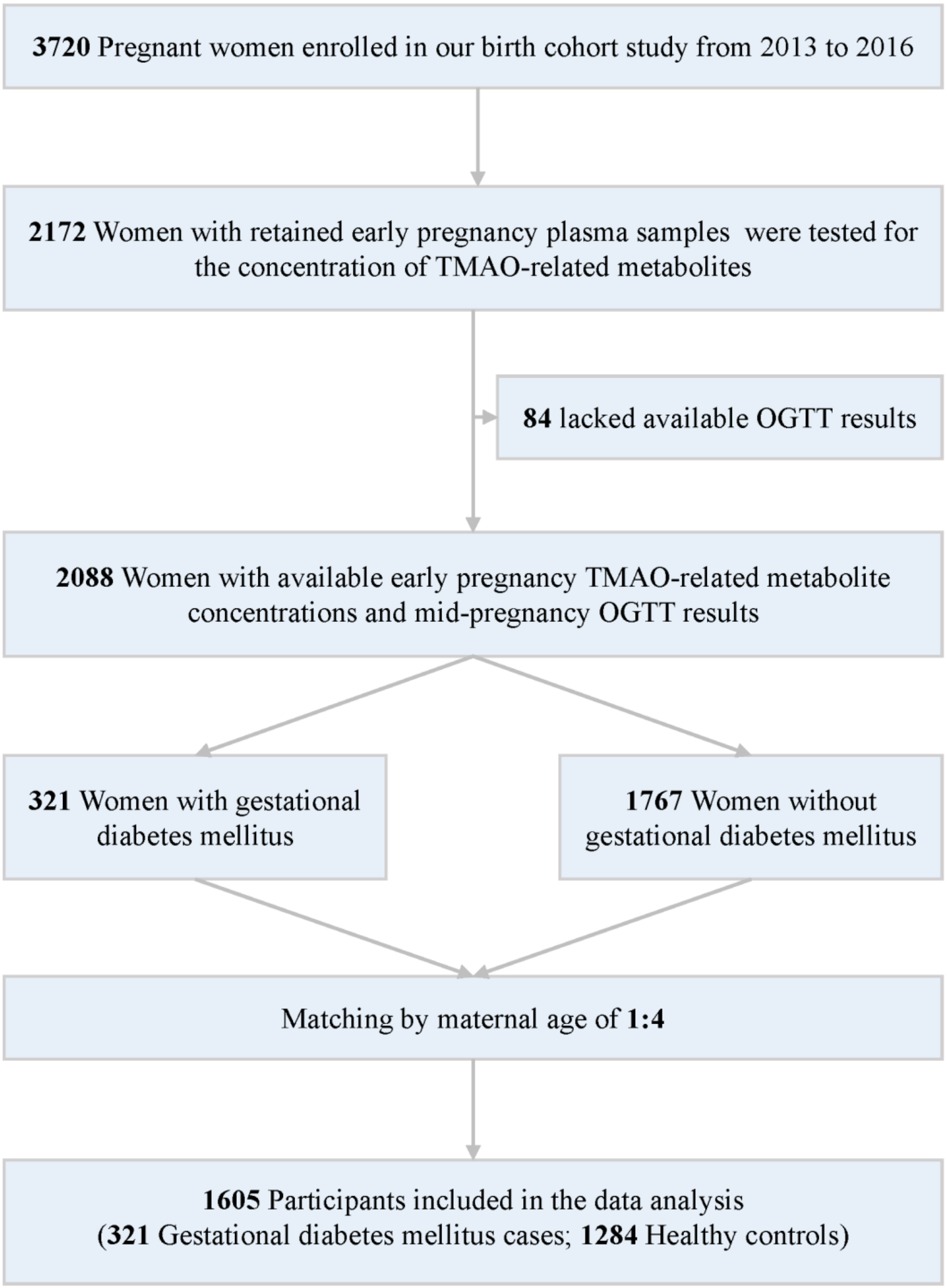

Figure S2. Flow Chart of the Study Participants' Selection

Abbreviations: TMAO, trimethylamine N-oxide; OGTT, oral glucose tolerance test.

Table S1. Spearman partial correlation coefficients (ρ).

P \ ρ	TMAO	Choline	Betaine	Carnitine
**TMAO**	**1.00**	0.11 ***	0.17 ***	-0.03 NS
**Choline**	*P* <0.001	1.00	0.14 ***	0.35 ***
**Betaine**	*P* <0.001	*P* <0.001	1.00	-0.03 NS
**Carnitine**	*P* =0.111	*P* <0.001	*P* =0.211	1.00

Spearman partial rank-order correlation of plasma metabolite levels. Abbreviations: TMAO, Trimethylamine N-oxide; NS, no significance (*P* >0.05); ***, *P* <0.001.

Table S2. Participant characteristics in sensitivity analysis.

	Overall (N=1605)	Controls (N=1284)	GDM Cases (N=321)	*P*
**Prenatal characteristics**				
Age (years)	29.7 ± 5.2	29.7 ± 5.2	29.9 ± 5.5	**0.005**
Ethnicity				0.899
Han	1589 (99.0)	1271 (99.0)	318 (99.1)	
Others	16 (1.0)	13 (1.0)	3 (0.9)	
Maternal education				0.184
High school and below	158 (9.8)	122 (9.5)	36 (11.2)	
College	364 (22.7)	286 (22.3)	78 (24.3)	
University	891 (55.5)	724 (56.4)	167 (52.0)	
Postgraduate degree	192 (12.0)	152 (11.8)	40 (12.5)	
Pre-pregnancy BMI (kg/m^2)	21.10 [19.50, 23.30]	20.90 [19.30, 22.90]	22.00 [20.20, 24.70]	**<0.001**
Early-pregnancy BMI (kg/m^2)	22.00 [20.30, 24.40]	21.80 [20.20, 23.92]	23.40 [21.10, 26.20]	**<0.001**
Physical activity				0.643
Low	714 (44.5)	577 (44.9)	137 (42.7)	
Middle	852 (53.1)	674 (52.5)	178 (55.5)	
High	39 (2.4)	33 (2.6)	6 (1.9)	
Smoke	41 (2.6)	32 (2.5)	9 (2.8)	0.749
Alcohol consumptions	209 (13.0)	166 (12.9)	43 (13.4)	0.825
Live with smokers	585 (36.4)	471 (36.7)	114 (35.5)	0.688
Early pregnancy sleep	4.00 [3.00, 6.00]	4.00 [3.00, 6.00]	4.00 [3.00, 6.00]	0.133
Parity				0.304
Nulliparous	978 (60.9)	787 (61.3)	191 (59.5)	
Parous	627 (39.1)	497 (38.7)	130 (40.5)	
History of gestational hypertension	4 (0.2)	3 (0.2)	1 (0.3)	0.803
History of GDM	18 (1.1)	5 (0.4)	13 (4.0)	**<0.001**
History of hypertension	3 (0.2)	0 (0.0)	3 (0.9)	0.994
History of hyperlipidemia	15 (0.9)	9 (0.7)	6 (1.9)	0.059
Other disease	371 (23.1)	314 (24.5)	57 (17.8)	**0.010**
TMAO (μmol/L)	1.98 [1.17, 3.23]	1.97 [1.16, 3.23]	2.01 [1.21, 3.23]	0.511
Choline (μmol/L)	9.26 [7.28, 12.11]	9.23 [7.20, 12.11]	9.40 [7.64, 11.97]	0.276
Betaine (μmol/L)	20.51 [16.77, 24.71]	20.62 [17.06, 24.91]	19.10 [15.74, 23.42]	**0.005**
Carnitine (μmol/L)	17.04 [13.30, 20.96]	17.27 [13.41, 21.18]	16.28 [13.07, 20.44]	0.108
FPG (mmol/L)	4.40 [4.17, 4.70]	4.31 [4.10, 4.60]	4.80 [4.40, 5.20]	**<0.001**
1h PG (mmol/L)	7.70 [6.70, 8.80]	7.40 [6.50, 8.29]	10.00 [8.60, 10.60]	**<0.001**
2h PG (mmol/L)	6.50 [5.70, 7.40]	6.30 [5.60, 7.00]	8.50 [7.30, 9.10]	**<0.001**
TG (mmol/L)	1.46 [1.12, 2.24]	1.40 [1.10, 2.19]	1.66 [1.24, 2.57]	**<0.001**
TC (mmol/L)	4.70 [4.20, 5.36]	4.68 [4.17, 5.30]	4.84 [4.28, 5.55]	**0.018**
HDL (mmol/L)	1.19 [1.01, 1.45]	1.18 [1.01, 1.44]	1.22 [1.04, 1.55]	**0.004**
ALT (mmol/L)	14.00 [9.00, 23.00]	13.00 [9.00, 22.00]	16.00 [10.00, 25.00]	**0.034**
AST (mmol/L)	17.00 [14.00, 21.00]	17.00 [14.00, 20.00]	17.00 [14.00, 22.00]	0.159

Data are shown as n(%), mean ± SD, or median [IQR].

The comparison of characteristics between GDM cases and non-GDM controls was conducted using univariate conditional logistic regression. Statistically significant results are bolded.

Abbreviations: BMI, body mass index; GDM, gestational diabetes mellitus, TMAO, trimethylamine N-oxide; FPG, fasting plasma glucose; PG, plasma glucose; TG, triglycerides; TC, total cholesterol; HDL, High-density lipoprotein; ALT, alanine aminotransferase; AST, aspartate aminotransferase.

Table S3. Associations between maternal trimethylamine N-oxide (TMAO) and its precursor concentrations in early pregnancy and the risk of GDM in mid-pregnancy in sensitivity analysis.

	**Crude Model**		**Multivariable Adjusted Model [a]**	
	**OR (95% CI) [b]**	***P***	**OR (95% CI)**	***P***
**TMAO (μmol/L)**				
Q1 (≤1.17)	Ref.		Ref.	
Q2 (1.17-1.98)	1.15 (0.81, 1.63)	0.426	1.23 (0.84, 1.80)	0.293
Q3 (1.98-3.23)	1.10 (0.78, 1.56)	0.581	1.06 (0.73, 1.56)	0.752
Q4 (>3.23)	1.08 (0.76, 1.53)	0.667	0.95 (0.64, 1.41)	0.808
Continuous [c]	0.96 (0.84, 1.09)	0.511	0.90 (0.77, 1.06)	0.200
**Choline (μmol/L)**				
Q1 (≤7.28)	Ref.		Ref.	
Q2 (7.28-9.26)	1.33 (0.93, 1.89)	0.118	1.32 (0.89, 1.95)	0.167
Q3 (9.26-12.11)	1.36 (0.95, 1.95)	0.095	1.46 (0.97, 2.18)	0.067
Q4 (>12.11)	1.19 (0.83, 1.71)	0.336	1.23 (0.80, 1.89)	0.338
Continuous	1.07 (0.95, 1.21)	0.276	1.09 (0.95, 1.25)	0.224
**Betaine (μmol/L)**				
Q1 (≤16.77)	Ref.		Ref.	
Q2 (16.67-20.51)	**0.71 (0.51, 0.99)**	**0.042**	0.75 (0.52, 1.08)	0.126
Q3 (20.51-24.71)	0.74 (0.52, 1.04)	0.078	0.74 (0.51, 1.09)	0.124
Q4 (>24.71)	**0.51 (0.35, 0.74)**	**<0.001**	**0.56 (0.37, 0.85)**	**0.006**
Continuous	**0.82 (0.71, 0.94)**	**0.005**	**0.85 (0.73, 0.99)**	**0.033**
**Carnitine (μmol/L)**				
Q1 (≤13.30)	Ref.		Ref.	
Q2 (13.30-17.04)	1.00 (0.72, 1.40)	0.980	0.87 (0.60, 1.27)	0.473
Q3 (17.04-20.96)	0.75 (0.53, 1.08)	0.123	**0.65 (0.43, 0.98)**	**0.038**
Q4 (>20.96)	**0.68 (0.47, 0.99)**	**0.043**	**0.55 (0.36, 0.85)**	**0.007**
Continuous	0.90 (0.79, 1.02)	0.108	**0.84 (0.72, 0.98)**	**0.027**

a. Multivariable adjusted model has adjusted for age, race, educational level, early pregnancy BMI, physical activity, smoking, alcohol, passive smoke, early pregnancy sleep score, pregnancy history, disease history and the levels of other three metabolites.
b. Conditional logistic regression was used to estimate the associations. The effect estimate was expressed as odds ratios (OR) and 95% confidence interval (95% CI). Statistically significant results are bolded.
c. "Continuous" refers to the increase of per standard deviation (SD).

Table S4. Associations between maternal concentrations of trimethylamine N-oxide (TMAO) and its precursors in early pregnancy and glycemic indicators in mid-pregnancy in sensitivity analysis.

	FPG		1h-PG		2h-PG	
	**β (95% CI) [a]**	***P***	**β (95% CI)**	***P***	**β (95% CI)**	***P***
**TMAO (μmol/L)**						
Q1 (≤1.17)	Ref.		Ref.		Ref.	
Q2 (1.17-1.98)	0.02 (-0.05, 0.09)	0.556	0.14 (-0.09, 0.37)	0.235	0.03 (-0.17, 0.23)	0.734
Q3 (1.98-3.23)	0.03 (-0.04, 0.1)	0.441	0.00 (-0.23, 0.23)	0.994	-0.06 (-0.26, 0.14)	0.542
Q4 (>3.23)	0.04 (-0.03, 0.12)	0.254	0.11 (-0.12, 0.34)	0.339	0.04 (-0.17, 0.24)	0.722
Continuous [b]	0.01 (-0.01, 0.04)	0.289	0.03 (-0.05, 0.11)	0.515	0.02 (-0.05, 0.09)	0.519
**Choline (μmol/L)**						
Q1 (≤7.28)	Ref.		Ref.		Ref.	
Q2 (7.28-9.26)	0.04 (-0.03, 0.12)	0.254	0.14 (-0.09, 0.37)	0.220	0.06 (-0.14, 0.27)	0.532
Q3 (9.26-12.11)	0.07 (-0.01, 0.14)	0.079	**0.35 (0.11, 0.58)**	**0.004**	0.19 (-0.01, 0.4)	0.063
Q4 (>12.11)	0.01 (-0.07, 0.09)	0.803	0.23 (-0.01, 0.48)	0.066	0.08 (-0.13, 0.30)	0.460
Continuous	-0.01 (-0.04, 0.02)	0.482	**0.09 (0.01, 0.18)**	**0.031**	0.05 (-0.03, 0.12)	0.229
**Betaine (μmol/L)**						
Q1 (≤16.77)	Ref.		Ref.		Ref.	
Q2 (16.67-20.51)	-0.01 (-0.08, 0.06)	0.811	-0.08 (-0.31, 0.15)	0.483	-0.03 (-0.23, 0.17)	0.789
Q3 (20.51-24.71)	-0.04 (-0.12, 0.03)	0.232	-0.02 (-0.25, 0.21)	0.878	-0.14 (-0.34, 0.07)	0.187
Q4 (>24.71)	**-0.08 (-0.16, -0.01)**	**0.031**	**-0.27 (-0.51, -0.04)**	**0.023**	**-0.27 (-0.48, -0.06)**	**0.011**
Continuous	-0.03 (-0.05, 0. 00)	0.049	-0.08 (-0.16, 0.00)	0.056	**-0.08 (-0.15, -0.01)**	**0.026**
**Carnitine (μmol/L)**						
Q1 (≤13.30)	Ref.		Ref.		Ref.	
Q2 (13.30-17.04)	0.02 (-0.06, 0.09)	0.660	0.03 (-0.19, 0.26)	0.776	-0.03 (-0.23, 0.17)	0.800
Q3 (17.04-20.96)	-0.04 (-0.11, 0.04)	0.349	-0.15 (-0.39, 0.09)	0.211	-0.21 (-0.41, 0.00)	0.053
Q4 (>20.96)	0.04 (-0.04, 0.12)	0.313	-0.11 (-0.36, 0.13)	0.360	-0.2 (-0.42, 0.01)	0.061
Continuous	**0.03 (0.00, 0.05)**	**0.048**	-0.03 (-0.12, 0.05)	0.461	**-0.08 (-0.15, 0.00)**	**0.047**

a. Multivariate linear regression was used to estimate the associations, adjusting for age, race, educational level, early pregnancy BMI, physical activity, smoking, alcohol, passive smoke, early pregnancy sleep score, pregnancy history, disease history and the levels of other three metabolites. The effect estimate was expressed as Beta coefficient (β) and 95% confidence interval (95% CI). Statistically significant results are bolded.
b. "Continuous" refers to the increase of per standard deviation (SD).

Abbreviations: BMI, body mass index; TMAO, trimethylamine N-oxide; FPG, fasting plasma glucose; 1h PG, 1-hour plasma glucose; 2h PG, 2-hour plasma glucose.
